# Exploring the Multi–Target Neuroprotective Chemical Space of Benzofuran Scaffolds: A New Strategy in Drug Development for Alzheimer’s Disease

**DOI:** 10.3389/fphar.2019.01679

**Published:** 2020-01-31

**Authors:** Jaime R. Cabrera-Pardo, Jorge Fuentealba, Javiera Gavilán, Daniel Cajas, José Becerra, Mariola Napiórkowska

**Affiliations:** ^1^ Departamento de Química, Facultad de Ciencias, Universidad del Bio-Bio, Concepción, Chile; ^2^ Departamento de Botánica, Facultad de Ciencias Naturales y Oceanográficas, Universidad de Concepción, Concepción, Chile; ^3^ Department of Chemistry, University of Utah, Salt Lake City, Utah, United States; ^4^ Departamento de Fisiología, Facultad de Ciencias Biológicas, Universidad de Concepción, Concepción, Chile; ^5^ Chair and Department of Biochemistry, Medical University of Warsaw, Warsaw, Poland

**Keywords:** Alzheimer’s disease, benzofuran, natural products, chemical libraries, Andean-Patagonian fungi

## Abstract

Alzheimer’s disease (AD) is an irreversible and progressive neurodegenerative disorder that slowly destroys memory. The precise mechanism of AD is still not entirely understood and remains under discussion; it is believed to be a multifactorial disease in which a number of mechanisms are involved in its pathogenesis. Worldwide, near 37 million people suffer from the effects of AD. As a cause of death for elderly, it is predicted that AD will rank third in the coming years, just behind cancer and heart disease. Unfortunately, AD remains an incurable condition. Despite the devastating problems associated with AD, there are only four FDA approved drugs for palliative treatment of this pathology. Hence, renewed scientific efforts are required not only to uncover more insights into the AD process but also to develop more efficient pharmacological tools against this disease. Due to the complexity and multiple mechanisms at play in the progression of AD, the development of drugs by rational design is extremely difficult. The existing drugs to fight against Alzheimer’s have had limited success, mainly due to their ability to modulate only one of the mechanisms involved in AD. As opposed to single-targeted strategies, the identification of small molecules able to affect multiple pathways involved in Alzheimer’s is a promising strategy to develop more efficient medicines against this disease. Central to existing efforts to develop pharmaceuticals controlling AD is the discovery of new chemicals displaying strong neuroactivity. Benzofurans are privileged oxygen containing heterocycles that have a strong neuroprotective behavior, inhibiting several of the important events involved in the AD process. In this review, an approach is presented that relies on expanding the neuroprotective chemical space of benzofuran scaffolds by accessing them from Andean–Patagonian fungi and synthetic sources (chemical libraries). The exploration of the neuroprotective chemical space of these scaffolds has the potential to allow the discovery of substitution patterns that display multi-target neuroactivity against multiple events involved in AD. This benzofuran chemical framework will establish a multi-target chemical space that could set the basis for the development of super drugs against AD.

## Introduction

Alzheimer’s disease (AD), the most common cause of dementia, is a neurodegenerative disorder that affects areas of the cerebral cortex and hippocampus (Hane et al., 2017). Abnormalities, usually first detected in the frontal and temporal lobes, then slowly and unremittingly progress to other areas of the neocortex (Masters et al., 2015). The precise mechanism of AD is still not entirely understood and it is believed to be a multifactorial disease in which a number of mechanisms are involved in its pathogenesis (Robinson et al., 2017). Since the initial report identifying an amyloid protein in postmortem patients and the amyloid cascade hypothesis in the early 1990’s, efforts to investigate AD have grown exponentially. Currently, studies regarding AD reach over 20,000 articles (Hane et al., 2017). While all these studies have certainly contributed to understanding AD, there is still a long path ahead and much to uncover in order to fully comprehend this multifactorial and devastating disease.

Improvements in medical care and life style have led to a substantial growth in the elderly population (Wimo et al., 2003). Due to increasing lifespans and the close association of AD with aging, this disease may become an intractable problem on a global scale in the near future. Worldwide, nearly 37 million people are currently affected by dementia and the majority is AD related (Wimo et al., 2003; Qiu et al., 2009). This figure is already increasing at a disturbing pace; around 6 million new cases of AD are diagnosed per year in the geriatric population. In 2013, nearly 85,000 deaths from AD were recorded, positioning AD as the sixth leading cause of death in the United States (Wimo et al., 2003; Qiu et al., 2009). Globally, costs related to AD treatment reached approximately US$315 billion back in 2005 (Qiu et al., 2009). These costs are expected to double by 2040 (Hurd et al., 2013).

Unfortunately, AD remains an incurable condition. Existing drugs approved by the U.S. Food and Drug Administration (FDA) against AD only ameliorate its symptoms. Despite the devastating problems associated with AD, there are only four FDA approved drugs for the treatment of this disorder (donepezil, galantamine, rivastigmine, and memantine). Shockingly, the pace of production of new drugs to control Alzheimer’s is stagnating, without any new entity incorporated into the pharmacological arsenal during the last 15 years (Casey et al., 2010; Mullard, 2012; Becker et al., 2014; Cummings et al., 2017). Hence, scientific efforts are required not only to uncover more insights into the AD process but also to develop more efficient pharmaceuticals against this disease. Central to existing efforts to develop medicines controlling AD is the identification of chemical cores displaying strong neuroactivity (Aziz et al., 2014). Due to the complexity of the AD process, involving a number of different mechanisms, the development of drugs by rational design has proved cumbersome (Crews and Masliah, 2010). As opposed to single-targeted strategies, the identification of small molecules able to modulate multiple pathways involved in Alzheimer’s can result in the development of more efficient medicines against this disease (Bajda et al., 2011; Leon et al., 2013; Dias et al., 2014; Prati et al., 2016; Unzeta et al., 2016). Thus, taking into consideration the chemical space that is relevant in biological systems, herein we present an approach for the identification and investigation of a region of this space with neuroprotective bioactivity, potentially leading to the discovery of molecules displaying a multi-target behavior. We envision that this could be achieved by taking advantage of the rich chemistry provided by natural and synthetic sources.

## Neuroprotective Chemical Space

Organic molecules are defined by a number of variables including connectivity, type, number, and stereochemistry of the atoms described in the structural formula. Taking into account these descriptors, plus the molecular weight and fundamental laws of physical organic chemistry, there is a definable number of small organic molecules that can be synthesized. This is the chemical space, which is an important concept in cheminformatics, and refers to the set of all small organic molecules from natural or synthetic origins (Reymond, 2015). Some have estimated chemical space to reach beyond 10^60^; a number that despite being finite, is preposterously high and inaccessible to explore by existing synthetic methods. The development of small molecules that control protein function is at the center of chemical biology research and the pharmaceutical industry. Crucial to the discovery of these important compounds is the identification of the biologically relevant chemical space, which is defined as the set of chemical cores that are required for biological systems to operate (Dobson, 2004; Koch et al., 2005; Reymond et al., 2010; Deng et al., 2013). For instance, the total number of small organic molecules present in the human body reaches a few thousand. As a consequence, the biologically relevant chemical space represents only a fraction of the total chemical space and is of great importance for the identification of molecules with interesting biological activity.

While the biologically relevant chemical space refers to the ensemble of small molecules crucial for biological systems to function, it is important to identify a subset of this space that displays a given biological activity. In living organisms, the continuing evolution of biosynthetic routes has equipped natural products with scaffolds that act as modulators of biochemical pathways by effectively binding to proteins. Alternatively, in the laboratory, synthetic methodology has grown tremendously during the last century, allowing practitioners of medicinal chemistry to assemble biomimetic cores able to modulate biological systems. Taking into consideration these sources of bioactive molecules, the ensemble of compounds originating from both natural and synthetic origins displaying a particular biological effect is defined as the bioactive chemical space (Klenner et al., 2012; Von Salm et al., 2015; Zhang et al., 2015; Bajorath, 2016; Prati et al., 2016; Mei et al., 2017). As part of the drug development process, it is paramount to identify and explore this biologically active space since it narrows the universe of potentially active molecules, accelerating the pace of the discovery of new medicines. Thus, identifying and exploring parts of the biologically active chemical space with neurological effects could lead to the discovery of compounds displaying neuroprotective and multi-target bioactivities.

## Benzofuran Scaffolds Are Promising Targets to Explore the Neuroprotective Chemical Space

**Figure 1:**
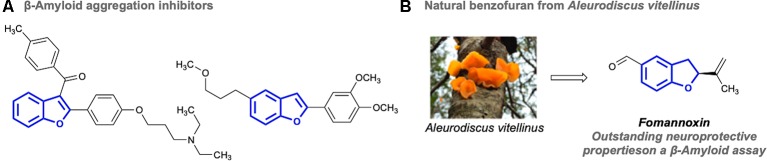
Some benzofuran scaffolds with neuroprotective AD bioactivities. **(A)** Examples of synthetic benzofurans with inhibitory properties against beta-amyloid. **(B)** A natural benzofuran isolated from Aleurodiscus vitellinus.

Benzofurans are privileged oxygen containing heterocycles that are present in a number of biologically important molecules. This class of chemical motifs have been shown to display antibacterial, antifungal, antioxidant, antitumoral, antiinflammatory, anticonvulsant, and anti-HIV bioactivities (Hiremathad et al., 2015; Khanam, 2015; Nevagi et al., 2015). Interestingly, benzofurans have also been found to have neuroprotective activity, inhibiting the important events involved in the AD process (Howlett et al., 1999; Allsop et al., 2001; Ono et al., 2002; Ono et al., 2006; Rizzo et al., 2008; Rizzo et al., 2012; Sashidhara et al., 2014; Ha et al., 2017; González-Ramírez et al., 2018; Kumar et al., 2018). Our research group has also investigated the role of natural fungal benzofurans in synaptic failure, decay of intracellular Ca^2+^ transients and synaptic disorganization (González-Ramírez et al., 2018). Although several factors are involved in the development of AD, it has been established that the production of toxic Aβ is crucial for the genesis of AD. Indeed, benzofuran scaffolds have been reported to play an important role as inhibitors of Aβ fibril formation (**Figure 1A**). Despite the promising neuroactivity displayed by benzofuran derivatives, their bioactivity still remains single-targeted in nature and a systematic study exploring the neuroprotective chemical space of these interesting heterocycles has not been carried out (González-Ramírez et al., 2018). This investigation is essential to find the chemical fundamentals that would provide the knowledge necessary to develop multi-target AD drugs based on benzofuran scaffolds. Existing reports show that the neuroprotective bioactivity of benzofuran derivatives falls into the µM range (Howlett et al., 1999; Allsop et al., 2001; Ono et al., 2002; Ono et al., 2006; Rizzo et al., 2008; Rizzo et al., 2012; Sashidhara et al., 2014; Ha et al., 2017; González-Ramírez et al., 2018). Thus, chemical probes containing benzofuran cores have yet to become approved drugs to control AD, mainly due to their modest potency. The lack of drug development could be attributed to the limited knowledge of protein-ligand interactions between benzofurans and important protein targets involved in AD progression (Howlett et al., 1999; Allsop et al., 2001; Kumar et al., 2018). In order to increase their efficacy, chemical transformation can be employed to tune their biological properties. Such chemical modifications could be achieved either by hemi-synthesis or total synthesis of natural benzofuran cores. In addition to blocking the Aβ fibril formation process, benzofuran compounds have proven to be efficient inhibitors of butyrylcholinesterase (Kumar et al., 2018), which is another important target for drug development to control AD. Given the importance of developing multitarget probes to control AD (Bajda et al., 2011; Leon et al., 2013; Prati et al., 2016; Unzeta et al., 2016), the dual activity displayed by benzofuran scaffolds against different mechanisms of AD make them worthwhile ligand targets for drug development. Thus, synthetic efforts in conjunction with the development of benzofuran based chemical libraries would provide the necessary collection of molecules to efficiently explore the multi-target neuroprotective chemical space of this scaffold, facilitating the discovery of effective pharmaceuticals against AD.

Chemical libraries, which can be inspired by natural products or synthetic scaffolds, are collections of chemicals often used in high-throughput screening (Shelat and Guy, 2007; Grabowski et al., 2008; Quinn et al., 2008; Galloway et al., 2010; Gu et al., 2013; Saleeb et al., 2018). The development of chemical libraries relies on well-known and robust chemical transformations in which practitioners of organic chemistry can generate a number of variations on a defined molecular scaffold or backbone. In the drug discovery process, a range of small molecules are synthesized and tested against biological assays. Efficient screening requires the synthesis of chemical libraries covering a desired biologically relevant chemical space. More specifically, the development of a collection of molecules focused on a bioactive chemical space delivers a number of variations on pharmaceutically privileged scaffolds, accelerating the drug discovery process.

## Fungal Benzofuran Natural Products Are a Prolific Source of Chemical Probes to Explore the Neuroprotective Chemical Space

Natural products display unique biological activities and are still the main source of medicines that propels the drug discovery field (Cragg and Newman, 2013; Newman and Cragg, 2016). Fungi represent one of the largest groups of organisms. They are widely distributed across mild and extreme ecosystems on our planet (Choi and Kim, 2017). In addition, these organisms are entirely heterotrophic as they are unable to perform photosynthesis. Consequently, they have developed a unique metabolic plasticity allowing them to rapidly adapt and survive through the biosynthesis of an array of fascinating natural products (Calvo et al., 2002). Recent analysis of fungal genomes has revealed a vast number of secondary metabolite pathways that can be tuned or modified, allowing the production of novel and useful chemical scaffolds (Nielsen et al., 2017). Fungi-derived natural products are pharmaceutically prolific, having been developed into a number of important biological applications ranging from highly potent toxins to approved drugs (Schueffler and Anke, 2014). Over the last decades, several natural products showing encouraging biological activities have been isolated from fungi. For example, in the course of the long–standing research program of natural product chemistry at the University of Concepcion (Chile), we have recently investigated the secondary metabolites of *Aleurodiscus vitellinus*, isolated from Chilean Patagonia (González-Ramírez et al., 2018). We discovered that fomannoxin, a natural benzofuran compound, showed outstanding neuroprotective properties according to an amyloid-β peptide model (**Figure 1B**). The importance of this finding lies in the fact that anti-amyloid therapies are considered to be a promising alternative to existing AD medicines. Considering that amyloid species, especially the oligomers, have been associated with early synaptic toxicity, controlling the aggregation process could represent an interesting approach to develop new pharmacological tools (Lal et al., 2007).

The multifactorial nature of AD requires treatment with molecules able to target multiple pathogenic events. The discovery of multi-target probes would enable the development of effective AD drugs that, to date, are not available. Given the promising potential of Andean-Patagonian fungi from our previous studies on *A. vitellinus*, the neuroprotective chemical space of benzofurans may be able to be effectively expanded by isolating more of these scaffolds from other fungi. This scientific exercise would potentially enable the discovery of fungal benzofurans displaying similar bioactivity as in our previous work: positive effects on neuronal functionality (González-Ramírez et al., 2018).

**Figure 2:**
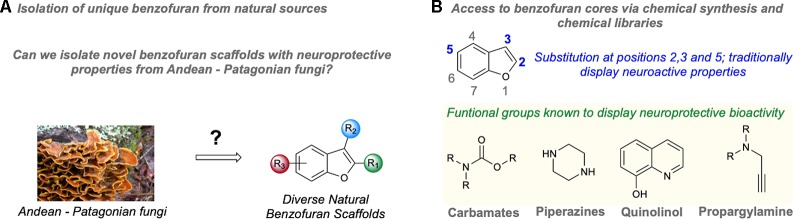
Strategies to expand the neuroprotective chemical space of benzofurans. **(A)** Schematic for the isolation of benzofuran scaffolds from natural fungal sources. **(B)** Schematic of synthetic strategies to access benzofurans.

While the Andean-Patagonian ecosystem displays a high chemical and biological diversity, mycological studies in this environment have been limited mainly due to challenges in accessibility and extreme weather conditions (Gamundí, 2003). This ecosystem displays unique microclimate and terrain conditions promoting high levels of endemism (Cowling et al., 1994). Thus, investigating the chemical diversity of fungi from Andean-Patagonian environments could lead to the discovery of highly diverse benzofuran scaffolds, expanding the neuroprotective chemical space of this heterocycle from natural sources (Youdim et al., 2014, **Figure 2A**).

## Chemical Libraries Based on Benzofuran Scaffolds Offer an Efficient Platform to Investigate the Neuroprotective Chemical Space

Studies investigating the neuroprotective activity of synthetic benzofurans have shown that chemical substitution at positions 2, 3, and 5 on this heterocycle results in compounds displaying neuroactivity (Howlett et al., 1999; Allsop et al., 2001; Ono et al., 2002; Ono et al., 2006; Rizzo et al., 2008; Rizzo et al., 2012; Sashidhara et al., 2014; Ha et al., 2017; González-Ramírez et al., 2018). Despite the ability of benzofuran cores to control important events in AD development, structure-activity relation studies on neuroprotection are limited (Wakabayashi et al., 2016), making the drug development process to control AD lengthy and cumbersome. To our knowledge, a systematic chemical study exploring the neuroprotective chemical space of benzofurans has not been carried out. In the development of other chemicals as treatments for neurological diseases, several chemical functionalities have been described that display strong neuroprotective behavior (**Figure 2B**). For instance, carbamates are the main player responsible for the bioactivity of rivastigmine, which is an acetylcholinesterase inhibitor used for the treatment of Alzheimer’s and Parkinson’s disease (Youdim et al., 2014). Piperazines have also displayed a range of biological activities including neuroprotective effects (Varadaraju et al., 2013; Modi et al., 2014; Wang et al., 2016). The quinolinol moiety is a strong radical scavenger present in Tacrine, the first drug approved for the treatment of AD (FernáNdez-Bachiller et al., 2010). Propargylamines are also neuroprotective functional groups by inhibiting MAO (Gal et al., 2005; Zheng et al., 2005). Thus, an interesting strategy would be the preparation of benzofuran hybrids containing the neuroactive functional groups identified in these studies, hoping to obtain benzofurans displaying positive synergistic effects on neuroprotective multi-target biological assays. This represents a rational strategy to achieve compounds displaying multi-target neuroprotective activity because it combines a neuroactive core linked to neuroactive functional groups, increasing the chances to access single molecules modulating multiple factors responsible for AD.

## Main Mechanisms Involved in Alzheimer’s Disease: Target to Develop Multitarget Chemical Probes

Approved FDA drugs to control AD are often ineffective since they target only one of the many possible routes that cause Alzheimer’s. Classic single-target chemical probes include: Acetylcholinesterase (AChase) (i.e. galantamine from *galantus sp*), NMDA receptors (memantine), oxidative stress (polyphenols: reseveratrol, catequins, antocianidins, etc), modulators of neuronal Acetylcholine receptors (nAChR, quinolizidinc alkaloids), or molecules that can interfere with the main event involved in the pathogenesis of disease, the amyloid β-peptide (βA) (Sala Frigerio and De Strooper, 2016; Cummings et al., 2017; Sharma et al., 2018). Moreover, during the last 15 years, no new drugs have been approved by the FDA for AD (Casey et al., 2010; Mullard, 2012; Becker et al., 2014; Cummings et al., 2017). Taking into consideration the stagnating production of therapeutics to control AD, the development of new targets against this disease is a valuable scientific niche that ought to be explored. It is important to note that chemicals developed to control targets for AD should reach promising levels of neuroprotective bioactivities so that they stand a chance to reach clinical use. Thus, new approaches to drug design are imperative, and the development of chemical probes capable of simultaneously targeting multiple mechanisms leading to AD is a highly promising route to effectively control this devastating disease. Several molecular mechanisms can be responsible for AD (Leon et al., 2013): deposition and aggregation of β-amyloid (oligomers and plaques); oxidative stress; cholinergic impairment; deregulation of calcium metabolism and metal dys-homeostasis; neuroinflammation; mitochondrial damage and hyperphosphorylated and β-folded tau proteins. Therefore, the development of chemical probes able to target multiple mechanisms involved in Alzheimer´s disease represents a distinct strategy to control this devastating disease.

## Conclusion

AD is a condition that still demands extensive efforts to understand its pathogenesis. Despite its devastating nature, AD is not curable and its symptoms can only be partially treated. Hence, the development of efficient pharmaceuticals against AD is in urgent need. The mode of action of existing FDA drugs relies on controlling single mechanisms of this pathology. While single-targeted approaches have been effective in certain pathologies, overall the use of this strategy to tackle multifactorial disease such as AD renders poor outcomes. For this reason, the development of drugs to control AD has stalled over the last decade. Current efforts to produce AD medicines have shifted towards the development of agents with multi-target activity in order to produce more effective pharmaceuticals. However, the generation of therapeutics that combine the biological effects of different AD drugs in a super molecule, modulating multiple events responsible for Alzheimer’s is extremely difficult. Nevertheless, benzofurans are heterocycles with promising neuroactive properties. We suggest that one promising approach is to expand the neuroprotective chemical space of benzofuran scaffolds by accessing these molecules from Andean–Patagonian fungi and synthetic sources. Exploring the neuroprotective chemical space of benzofuran scaffolds represents a distinct venue that could lead to the discovery of new substitution patterns displaying multi-target neuroactivity against multiple events involved in AD. This benzofuran chemical framework can establish a multi-target chemical space, setting the basis for the development of super drugs against AD.

## Author Contributions

JC-P conceived, designed and wrote the manuscript. JF and JG provided useful suggestions regarding the neuroprotective assays. DC and JB participated in the mycology aspect of the manuscript. MN gave input on the chemistry aspect of benzofurans.

## Funding

JC-P was kindly supported by FONDECYT regular 1190652. JF acknowledges the support of FONDECYT regular 1161078.

## Conflict of Interest

The authors declare that the research was conducted in the absence of any commercial or financial relationships that could be construed as a potential conflict of interest.
